# Large exchange bias in Cr substituted Fe_3_O_4_ nanoparticles with FeO subdomains†

**DOI:** 10.1039/d1nr04614d

**Published:** 2021-09-08

**Authors:** Claudiu Bulbucan, Calle Preger, Aram Kostanyan, Kirsten M. Ø. Jensen, Esko Kokkonen, Cinthia Piamonteze, Maria E. Messing, Rasmus Westerström

**Affiliations:** NanoLund, Lund University Box 118 22100 Lund Sweden; Synchrotron radiation research, Lund University SE-22100 Lund Sweden claudiu.bulbucan@sljus.lu.se; Solid State Physics, Lund University Box 118 22100 Lund Sweden; Physik-Institut, Universität Zürich CH-8057 Zürich Switzerland; Swiss Light Source, Paul Scherrer Institut CH-5232 Villigen PSI Switzerland; Department of Chemistry, University of Copenhagen Copenhagen Denmark; MAX IV Laboratory, Lund University PO Box 118 221 00 Lund Sweden

## Abstract

Tuning the anisotropy through exchange bias in bimagnetic nanoparticles is an active research strategy for enhancing and tailoring the magnetic properties for a wide range of applications. Here we present a structural and magnetic characterization of unique FeCr-oxide nanoparticles generated from seed material with a Fe : Cr ratio of 4.71 : 1 using a physical aerosol method based on spark ablation. The nanoparticles have a novel bimagnetic structure composed of a 40 nm ferrimagnetic Cr-substituted Fe_3_O_4_ structure with 4 nm antiferromagnetic Fe_*x*_O subdomains. Cooling in an applied magnetic field across the Néel temperature of the Fe_*x*_O subdomains results in a significant shift in the hysteresis, demonstrating the presence of a large exchange bias. The observed shift of *μ*_0_*H*_E_ = 460 mT is among the largest values reported for Fe_*x*_O–Fe_3_O_4_-based nanoparticles and is attributed to the large antiferromagnetic-ferrimagnetic interface area provided by the subdomains.

## Abstract 2

Here we present a structural and magnetic characterization of a novel bimagnetic nanoparticle composed of a 40 nm Cr-substituted magnetite phase with 4 nm FeO subdomains exhibiting large exchange bias.

## Graphical abstract

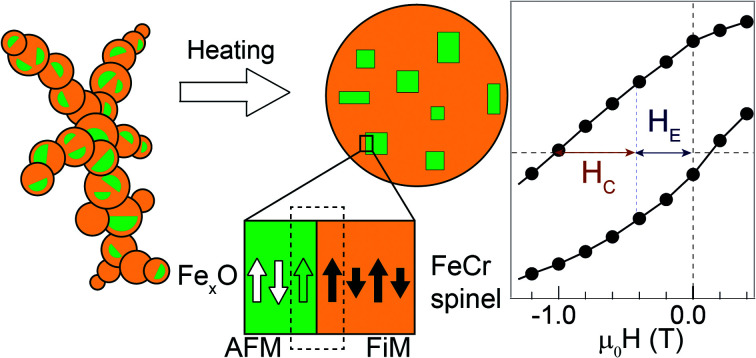

## 1. Introduction

Exchange bias (EB) in bimagnetic nanoparticles (NPs) composed of two differently ordered magnetic phases is an active research field motivated by the possibility of tuning the system's anisotropy for high-density data storage,^1^ spintronics,^2^ biomedical,^3–5^ and rare-earth-free permanent magnet applications.^6,7^ Bimagnetic NPs are commonly composed of a core–shell (CS) structure where the EB effect originates from the pinning of magnetic moments at the interface between an antiferromagnetic (AFM) and ferromagnetic (FM) or ferrimagnetic (FiM) phases when cooled in an applied magnetic field across the Néel temperature *T*_N_. The pinning of the interfacial magnetic moments leads to a unidirectional exchange anisotropy, making it more difficult to reverse the magnetization in the direction opposite the cooling field, and thus resulting in a horizontal shift (*H*_E_) in the hysteresis loop, oftentimes accompanied by an increase of the coercivity.^8,9^

An extensively studied class of bimagnetic systems are Fe-oxide NPs composed of an AFM wüstite Fe_*x*_O core and a FiM magnetite Fe_3_O_4_ shell.^5,10–21^ The most common approach for generating these systems is *via* thermal decomposition of Fe precursors, leading to metastable non-stoichiometric nano-crystalline Fe_*x*_O. Post-oxidation of the Fe_*x*_O nano-crystallites promotes diffusion and oxidation of Fe^2+^ into Fe^3+^ at the surface, leading to an inwards growth of the thermodynamically stable magnetite Fe_3_O_4_ spinel phase and the formation of Fe_*x*_O–Fe_3_O_4_ CS structures. Controlled post-synthesis treatment allows for varying the relative core-size dimensions, and studies have revealed a decreased coercivity and exchange-coupling with decreasing the size of the AFM phase due to a reduced AFM–FiM interface area and AFM anisotropy.^10,13,14^ Moreover, post-synthesis thermal treatment can lead to oxidation-induced anti-phase domain boundaries and atomic-scale defects, resulting in anomalous magnetic properties and EB effects in single-phase Fe_3_O_4_ NPs.^11,22^ Exchange-bias has also been observed in Zn^23^ and Co^7,24^ substituted Fe_*x*_O–Fe_3_O_4_ CS systems, where the latter results in a magnetically hard system with a record large shift of *μ*_0_*H*_E_ = 860 mT.

In bulk, high-temperature oxidation of Cr containing ferritic stainless steel leads to the formation of AFM Fe_*x*_O and Cr_2_O_3_ oxides, co-existing with FiM spinel oxides and the FM metallic FeCr phases.^25^ While the thin AFM oxides would have a negligible influence on the magnetic properties of the metallic FM bulk, the effect could be significant in nanoscale systems. However, studies of EB in Cr containing Fe-based bimagnetic NPs are scarce, and while there are a few based on metal–oxide CS systems^26,27^ and FeCr-oxides,^28^ they all exhibit modest EB, and to the best of our knowledge, there are no reports on Cr-substituted Fe_*x*_O–Fe_3_O_4_ based nanoscale systems.

We have previously reported on the generation of FeCr NPs using an aerosol technique based on spark ablation where the material is evaporated from stainless steel electrodes with a Fe : Cr ratio of 4.71 : 1. Depending on the carrier gas, both metallic and oxidized systems could be formed with a Fe to Cr ratio almost identical to the seed electrodes.^29^ This work focuses on the oxidized FeCr system and reports on the structural and magnetic characterization of NPs with an average diameter of 40 nm using a combination of magnetometry, electron microscopy, and synchrotron-radiation-based techniques. The NPs exhibit a novel structure composed of a 40 nm FeCr-spinel-oxide phase with 4 nm wüstite subdomains occupying up to 20% of the volume. In bulk stainless steel, Cr prevents the corrosion of metallic Fe by forming a passivating oxide layer that prevents the outward diffusion of iron ions and the inward diffusion of oxygen ions. We propose that Cr plays a similar role here but now stabilizing the wüstite subdomains in the generation process by forming a FeCr spinel-oxide that acts as diffusion barriers for the Fe^2+^ ions and preventing the complete conversion to the spinel phase. We demonstrate that the spinel structure is FiM and can be described as a Cr substituted magnetite phase with trivalent Cr ions occupying octahedral sites. The wüstite subdomains are AFM with a Néel temperature *T*_N_ ≈ 210 K and are believed to be Fe-rich with traces of Cr. The NPs are thus bimagnetic with a structure that we describe as a 40 nm FiM (Fe,Cr)_3_O_4_ spinel phase with 4 nm AFM Fe_*x*_O subdomains. The bimagnetic structure results in EB with a significant horizontal shift in the hysteresis loop at 2 K after cooling in a magnetic field from above the Néel temperature of the Fe_*x*_O subdomains. The shift of *μ*_0_*H*_E_ = 460 mT is one of the highest values reported for Fe_*x*_O–Fe_3_O_4_-based NPs and significantly larger than observed for CS systems with comparable size and similar Fe_*x*_O volume fraction.^12,18^ The large EB is attributed to the unique bimagnetic structure, which distributes the Fe_*x*_O phase throughout the particle volume, thereby increasing the surface to volume ratio and maximizing the interfacial area. The presented results demonstrate the potential of spark ablation techniques for generating novel bimagnetic NPs and indicate that the intra-particle distribution of the magnetic phases could provide an important parameter for tuning the exchange anisotropy and allowing for large EB effects while minimizing the amount of the AFM phase.

## 2. Experimental section and characterization

The NPs were generated by spark ablation^30–33^ described in detail for the present material system in a previous study.^29^ In short, a plasma channel between two stainless steel grade 430 electrodes with a Fe : Cr ratio of 4.71 : 1 evaporates material, which is transported away by a nitrogen carrier gas. Adiabatic expansion and mixing with the carrier gas cool down the vapor, which condensates into sub-10 nm primary particles that form larger agglomerates as they collide. The agglomerates are given a known charge before being transported through a furnace where they are compacted at a temperature of 1473 K and size-selected based on their electrical mobility. The charged NPs are finally deposited onto a substrate using an electric field. Moreover, if deposited in the presence of an applied magnetic field, the NPs can be guided to self-assemble into different multidimensional nano-structures.^34^

The structural characterization was achieved by means of X-ray diffraction (XRD) acquired at the 11-ID-B beamline at the Advanced Photon Source, Argonne National Laboratory.^35^ The X-ray photon wavelength was 0.2112 Å and the transmission scattering data was collected from NPs deposited on a glass slide. The NPs were characterized by a JEOL 3000F high-resolution transmission electron microscope (HRTEM) and the compositional analysis was performed with X-ray energy dispersive spectroscopy (XEDS) in scanning transmission electron microscopy (STEM) mode. The X-ray photoelectron spectroscopy (XPS) measurements were performed at the SPECIES beamline,^36^ MAX IV Laboratory, Lund, Sweden. The sample was deposited on Au coated single-crystalline Si wafers and the data acquisition was carried out under UHV conditions at room temperature. The X-ray absorption spectroscopy (XAS) data was measured at the X-treme beamline^37^ of the Swiss Light Source (SLS). The absorption spectra were acquired by measuring the total electron yield (TEY) in the on-the-fly mode^38^ while applying a magnetic field parallel to the X-ray beam. Simulated X-ray magnetic circular dichroism (XMCD) spectra were obtained using Cowan's Multiplet Structure software CTM4XAS.^39^ Temperature and field dependent magnetization data was recorded using a Quantum Design MPMS3 superconducting quantum interference device (SQUID) with a vibrating sample magnetometer (VSM) after depositing the particles onto a quartz substrate.

## 3. Results and discussion

### 3.1. Structural characterization

**Fig. 1:**
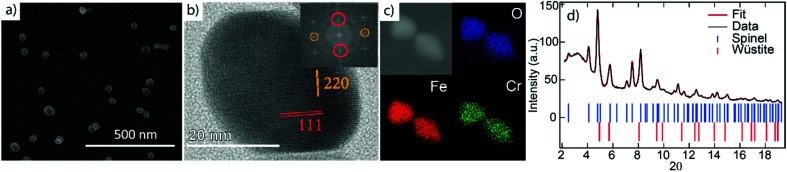
(a) Scanning electron microscopy (SEM) image of the particles; (b) transmission electron microscopy (HRTEM) image from a typical NP with the inclusion of the fast Fourier transform (FFT) analysis revealing a spinel structure; (c) energy dispersive X-ray spectroscopy (XEDS) showing homogeneous mixture of Fe, Cr and O; (d) XRD data with spinel and wüstite peaks.

**Fig. 2:**
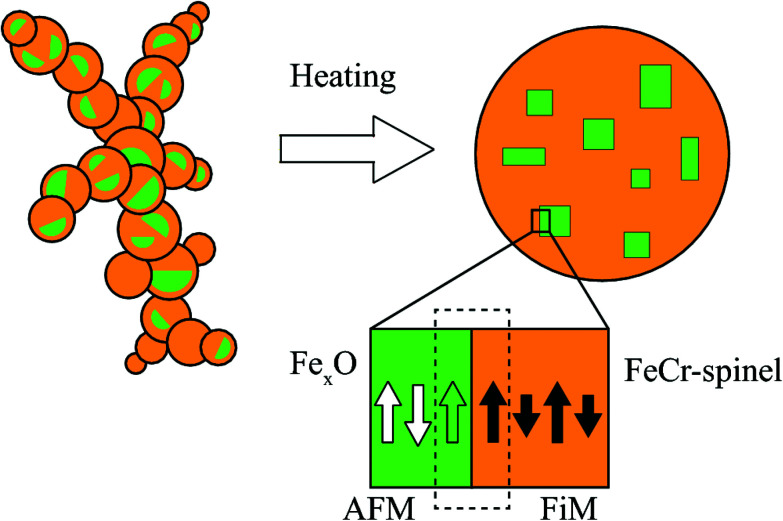
A schematic representation of an agglomerate composed of sub-10 nm primary particles that are compacted at 1473 K to produce a FiM FeCr-spinel structure with embedded AFM Fe_*x*_O subdomains. Also illustrated is the AFM–FiM interface, where cooling in an applied field across the Néel temperature leads to the pinning of magnetic moments at the boundary (dashed rectangle). The white and green arrows in the Fe_*x*_O subdomains represent the compensated and uncompensated spins, respectively.

The NPs imaged using scanning electron microscopy (SEM) in Fig. 1(a) exhibit visible facets and an average size of 〈*D*〉 ≈ 40 nm. Using HRTEM, the NPs appear single-crystalline with clear atomic lattice fringes corresponding to the {220} and {111} lattice planes of the spinel structure, consistent with our previous study^29^ (see Fig. 1(b)). Elemental mapping using XEDS reveals a homogeneous distribution of Fe, Cr, and O within the particles, indicating a FeCr-spinel-oxide. The 2*θ* scan in Fig. 1(d) exhibits Bragg peaks at positions corresponding to nanocrystalline spinel, consistent with the fast Fourier transform (FFT) of the HRTEM images, along with a large background signal originating from the glass slide. However, upon Rietveld refinement, a FeO (wüstite) rock salt structure had to be included to describe the data adequately. The refinements showed that the sample consists of about 80% spinel and 20% wüstite, and no other significant crystalline phases such as Cr_2_O_3_ were detected in the data. The average size of the rock salt crystallites is about one order of magnitude smaller 〈*D*〉 ≈ 4 nm than the spinel phase 〈*D*〉 ≈ 40 nm. The extracted lattice parameters *a*_r_ = 4.24 Å, and *a*_s_ = 8.36 Å obtained from the XRD analysis indicate a 1.2% and 0.4% compressed lattice for the rock-salt and spinel structure. The size of the spinel crystallites is in good agreement with the average particle diameters obtained using microscopy. Given that 4 nm particles do not reach the substrate, the wüstite phase should be contained within the ≈40 nm spinel structures, an assumption supported by magnetometry measurements. However, the volume fraction of 20% and the small size of the wüstite phase rule out a CS structure with Fe_*x*_O in the center. Instead, the results point to the formation of small Fe_*x*_O subdomains within the main spinel structure as schematically illustrated in Fig. 2.

**Fig. 3:**
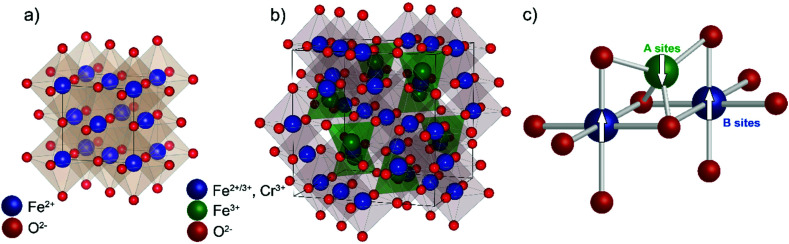
A schematic representation^40^ of the unit cell of stoichiometric FeO (a) and spinel (b), along with the occupied tetrahedral (A) and octahedral (B) interstitial sites of the O fcc lattice. (c) The FiM order of the (Fe,Cr)_3_O_4_ spinel with FM B–B and AFM A–B interactions. The arrows indicate the magnetic moment of the ions.

Wüstite and spinel share the same closed-packed fcc O-lattice, where each oxygen ion has one tetrahedral and two octahedral interstitial sites, referred to as the A and B sites respectively, see Fig. 3. The two structures differ only in the distribution of metal cations where stoichiometric wüstite has B-sites filled with divalent Fe ions while spinel has half of the B-sites and one-quarter of the A-sites occupied by ions with single or mixed valence. However, the diffusion of divalent ions in wüstite typically leads to a defective rock salt structure with vacancies compensated by two trivalent ions that can occupy both A and B-sites, resulting in the non-stoichiometric composition Fe_*x*_O (*x* = 0.83–0.96). Moreover, Fe_*x*_O is only metastable and transforms into the Fe_3_O_4_ spinel phase as Fe^2+^ ions diffuse and oxidize into Fe^3+^ at the surface. Given the similarities between the wüstite and spinel structures and the much smaller relative size of the former, it is not surprising that the local characterization of single NPs using HRTEM revealed a spinel structure since small defect rock salt clusters would be challenging to detect in the 2D projected image of the much larger spinel crystal structure.

The formation of the unique structure is attributed to the generation process and, importantly, the presence of Cr, which is known to influence the oxidation behavior of Fe significantly. In bulk, high-temperature oxidation of Cr containing ferritic stainless steel leads to the formation of Fe_*x*_O and spinel Fe-oxides. The corrosion resistance comes from the added Cr, which prevents the continuous oxidation of the metallic bulk by forming passivation layers of (Fe,Cr)_3_O_4_ spinel or Cr_2_O_3_ that hinder outward diffusion of Fe ions and inward diffusion of oxygen ions. Similar processes also occur on the nanoscale, where the exposure to ambient conditions can lead to the formation of a CS-structure with a passivating FeCr-oxide layer and a metallic core.^27,29^

However, it is important to note the difference to the present case where the NPs have no metallic phase and where the oxidation starts already in the formation of the primary particles and continues during the subsequent high-temperature compaction of the agglomerates.^29^ When the metal vapor condensates into primary particles in the presence of oxygen, the higher oxygen affinity of Cr could lead to an outwards diffusion and possibly CS-like structures with a Cr-enrichment towards the surface and predominantly Fe in the center. The resulting primary particles in the agglomerates would have local variations in the composition where the subsequent compactification at 1473 K would lead to further oxidation and redistribution of the elements. During this step in the generation process, the addition of Cr and the (Fe,Cr)_3_O_4_-spinel formation is expected to play a key role in stabilizing the Fe_*x*_O subdomains by acting as a diffusion barrier for the divalent Fe ions, thereby preventing the complete conversion into the (Fe,Cr)_3_O_4_-spinel phase.

### 3.2. XPS

**Fig. 4:**
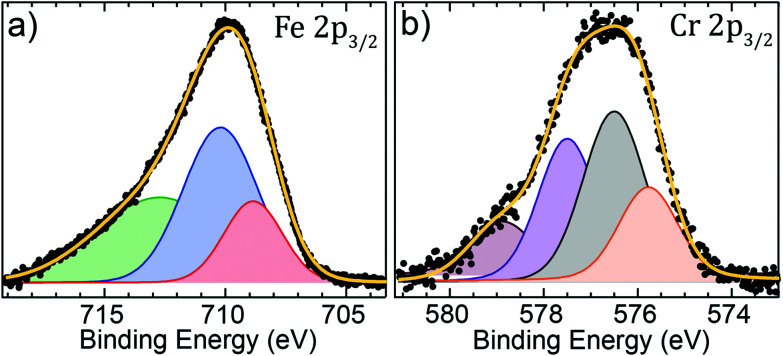
(a) Fe 2p_3/2_ core-level spectrum at a photon energy of 1150 eV; (b) Cr 2p_3/2_ core-level spectrum at a photon energy of 970 eV.

To complement the XEDS measurements, we measured XPS spectra from the Fe and Cr 2p core levels and the data is shown in Fig. 4. The absence of a clear satellite peak between the spin–orbit split Fe 2p peaks (see Fig. S1†) indicates that the system has a mixed-valence, and the 2p_3/2_ spectra can be deconvoluted using two main peaks corresponding to the Fe^2+^ ion residing in the B interstitial sites and the Fe^3+^ ions in both A and B sites.^41,42^ Furthermore, no metallic Fe peak could be identified from the data. Although the peak at 708.8 eV can be attributed to Fe^2+^, the octahedrally and tetrahedrally coordinated Fe^3+^ yield a complicated multiplet structure which cannot be assigned individually, but a Fe^3+^ : Fe^2+^ ratio of 2.43 : 1 was calculated from the fitted peak areas. An explanation for the Fe^2+^ deficiency could be the surface sensitivity of the technique, which probes the outer layers where Fe^2+^ ions are prone to oxidize into Fe^3+^.

Provided that Cr has a strong tendency of occupying octahedral sites with trivalent valence,^43,44^ the Cr 2p_3/2_ spectrum was decomposed using the four peaks found in the trivalent octahedrally coordinated chromite FeCr_2_O_4_ and chromium oxide Cr_2_O_3_, at binding energies of 575.8, 576.9, 577.8 and 578.9 eV.^45^ Moreover, no metallic Cr was detected. Spectra from the Fe and Cr 2p core levels were also recorded at higher photon energies, and the relative distribution of the two elements as a function of the kinetic energy of the photoelectrons is shown in Fig. S2.† The analysis reveals that the concentration of Cr increases with increasing kinetic energy, starting at only ≈7% and reaching ≈15% at the highest photon energy. Some reservations are needed for values extracted at the lower excitation energies since the photoelectrons are susceptible to diffraction effects at low kinetic energies, making the analysis unreliable. For the present system, we have demonstrated that the volume-averaged composition is close to that of 17.5% in the seed electrodes (Fe : Cr ratio of 4.71 : 1),^29^ and given the increase in the Cr concentration with increasing photon energy, we consider the XPS results consistent with previous measurements.

### 3.3. XMCD

**Fig. 5:**
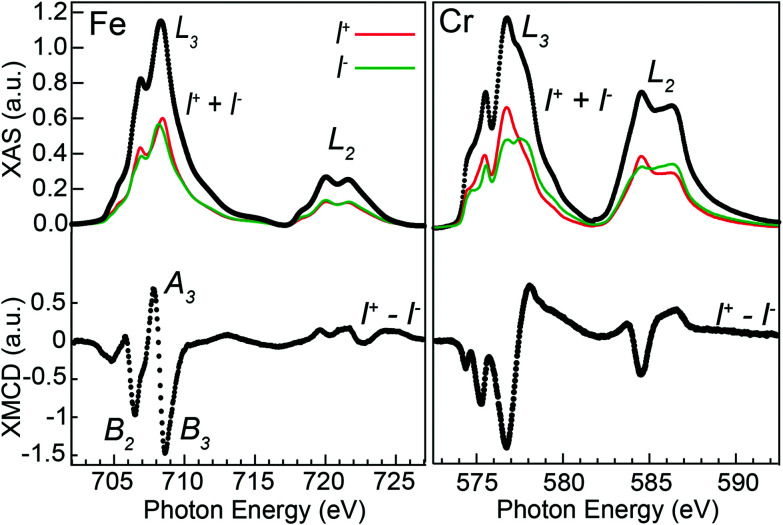
Top: polarization-dependent absorption using right (*I*^+^) and left (*I*^−^) circular polarized X-rays, and the total absorption (*I*^+^ + *I*^−^). Bottom: XMCD spectra obtained from the difference (*I*^+^ − *I*^−^).

Absorption measurements were performed using right (*I*^+^) and left (*I*^−^) circularly polarized X-rays at the Fe and Cr L_2,3_-edges (2p → 3d). The difference in absorption between the two helicities defines the XMCD spectra that probe the magnetic contribution of the 3d electrons of the absorbing ions and provide chemical and structural information. Fig. 5 shows polarization-dependent absorption and the corresponding XMCD spectra acquired in a 6.8 T magnetic field at the system's base temperature of about 2 K. The Fe XMCD spectrum in Fig. 5 (left-panel) shows resemblance to Fe_3_O_4_ ^46^ with three prominent peaks labeled B_2_, A_3_, and B_3_, corresponding to the individual contributions from the Fe_B_^2+^, Fe_A_^3+^, and Fe_B_^3+^ ions, respectively. Each contribution is proportional to the projection of the average magnetization of the absorbing ions onto the direction of the X-ray beam. Therefore, comparing the sign of the peaks’ maxima gives the average exchange coupling between the corresponding ions, and thus the FiM order of Fe_3_O_4_ with FM B–B and AFM A–B interactions. The Cr XMCD spectrum is similar to Fe_2_CrO_4_ ^47^ with Cr^3+^ in B sites, and the negative sign of the main peaks further demonstrate that the average Fe–Cr interaction within the B lattice is FM.

**Fig. 6:**
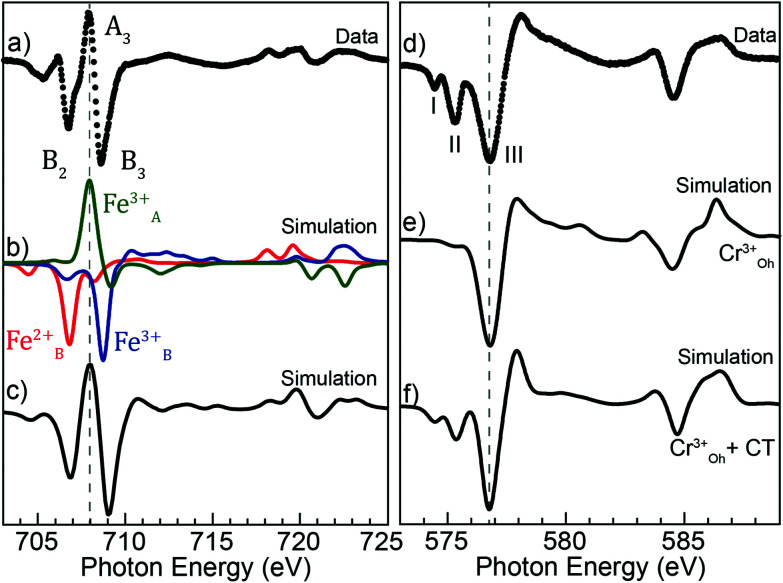
(a) The measured Fe L-edge XMCD spectrum; (b) multiplet simulations for each Fe ion; (c) weighted sum of the three Fe ions best reproducing the measured data; (d) the measured XMCD spectrum at the Cr L-edge; (e) Cr_B_^3+^ XMCD with no charge transfer; (f) Cr_B_^3+^ XMCD with charge transfer.

A more precise determination of the contributions to the different features is obtained by simulating the XMCD spectra using Cowan's Multiplet Structure software CTM4XAS.^39^ Given the spinel structure and the mixed Fe^2+^ and Fe^3+^ valence indicated by the spectroscopy measurements, the simulations were performed considering Fe_B_^2+^, Fe_B_^3+^, and Fe_A_^3+^ cations, as for Fe_3_O_4_. The d–d Slater integral reduction parameter was set to 0.87 (a value of 1 represents an 80% reduction) with the *F*_pd_ and *G*_pd_ kept at 1 as in previous works.^48–51^ Furthermore, crystal field splitting parameters 10*Dq* = 1.4 eV for the dication and 1.4 and −0.6 eV for the octahedral and tetrahedral Fe^3+^ were employed, along with exchange fields *gμ*_0_*H* = 0.1 and −0.1 eV for the B and A sites. The experimental XMCD spectra can be reproduced by forming a weighted linear sum of the individual contributions of the three cations and the best fit to the data is shown in Fig. 6(c).

For the inverse spinel structure of Fe_3_O_4_, schematic shown in Fig. 3, the ions would be equally distributed among the three sites, each contributing with a weight of 1/3 to the XMCD spectrum. For the present system, it is expected that Cr^3+^ occupies the B-sites, which would lower the relative weight of the corresponding di and trivalent Fe ions equally. However, the simulations reveal an increase of Fe_B_^3+^ (47%) and a decrease of Fe_B_^2+^ (20%). The probing depth at the Fe L_3_-edge when recording XMCD in TEY is about 4.5 nm in Fe_3_O_4_,^51^ and similar to the XPS measurements, the sensitivity to the outer layers where Fe^2+^ can oxidize into Fe^3+^ could partly explain the observed deficiency in Fe^2+^. However, the probing depth is considerably larger than for XPS, and importantly, the XMCD signal is proportional to the magnetic moments of the absorbing ions. Measurements performed at room temperature (see Fig. S4†) reveal an increased contribution from Fe^2+^, yielding a Fe^3+^ : Fe^2+^ ratio of 2.4 : 1, in excellent agreement with the ratio determined from the XPS analysis. The reduction in divalent Fe at 2 K can thus be explained by detecting Fe_*x*_O subdomains that predominantly have Fe^2+^ ions, which are paramagnetic and can align with the field at room temperature, but AFM coupled below the Néel temperature of 198 K and not contributing to the XMCD signal.

The simulations at the Cr edge were carried out by considering Cr^3+^ in B-sites. The 10*Dq* parameter was set to 1.5 (ref. 52) and the rest of the CTM4XAS parameters were kept at the same values as for the Fe simulations. The resulting simulated spectrum is shown in Fig. 6(f), and the measured Cr XMCD spectrum is given in Fig. 6(d). As can be observed, peaks denoted by I and II are not reproduced in the simulated spectrum, and to account for the discrepancy we considered charge transfer from the O^2−^ ligand to the Cr^3+^ d orbitals. The best match was achieved with a *Δ* = 0 suggesting a 1 : 1 mixture of 2p^6^3d^3^ and 2p^6^3d^4^ in the ground state of Cr^3+^ and, as can be observed in Fig. 6(e), peaks I and II are better reproduced by including charge transfer in the calculations. A more detailed description of the process and parameters chosen is given in the ESI.†

### 3.4. Magnetic properties

**Fig. 7:**
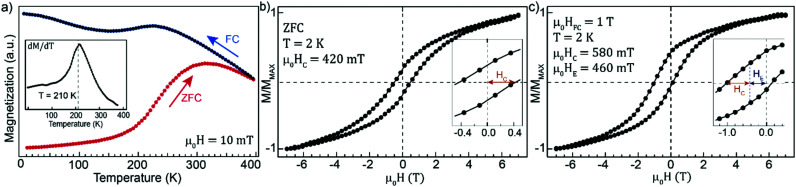
(a) Magnetization as a function of temperature measured at 10 mT; inset shows the derivative of the ZFC curve with respect to temperature; (b) magnetization curve after ZFC to 2 K; (c) magnetization curve after FC in a field of 1 T down from 400 K to 2 K.

Temperature-dependent magnetization measurements performed using SQUID magnetometry are shown in Fig. 7(a). The sample was zero-field cooled (ZFC) to 2 K, where a small field of 10 mT was applied, and the magnetization was recorded upon warming. Initially, the magnetization increases slowly up to a temperature of around 200 K, where a sharp increase is observed. The first derivative of the magnetization with respect to temperature reveals that the rate of the increase has a maximum around 210 K, which approximately coincides with the Néel temperature of *T*_N_ = 198 K of wüstite and can be explained by an AFM to paramagnetic transition of the Fe_*x*_O subdomains. The magnetization reaches a maximum at ≈320 K, after which it decreases and starts to merge at temperatures close to the measurement limit with the field-cooled (FC) curve recorded under the same initially applied field. The behavior is characteristic for super-paramagnetic systems where the maximum of the ZFC curve is associated with a blocking temperature at which the thermal energies become comparable to the anisotropy barrier. The magnetization of the FC curve increases up to around 200 K, where a small decrease is observed after crossing the Néel temperature. However, the decrease is only minor, and the magnetization continues to increase with decreasing temperature, after the initial drop. The small decrease in magnetization and the subsequent increase can be understood from the relatively small volume fraction of the Fe_*x*_O subdomains compared to the host spinel structure. Furthermore, the structure in Fig. 2 has a large AFM–FiM interface area and can be expected to have a significant number of uncompensated moments at the boundaries of the Fe_*x*_O subdomains, that can align with the field and contribute to the paramagnetic response. Observing the magnetic transition temperature *T*_N_ for ≈4 nm AFM subdomains is noteworthy given that the size is much smaller than the Fe_*x*_O core-sizes commonly reported for CS NPs.^18,53^ An exception is the Co substituted Fe_*x*_O–Fe_3_O_4_ system where *T*_N_ was observed for similar-sized AFM cores and explained by the large magnetocrystalline anisotropy of the mixed monoxide, making it less prone to size effects.^22^ It has been demonstrated that Cr substitution in Mn_3_O_4_ enhances *T*_N_,^54^ but the effect on Fe_*x*_O has, to the best of our knowledge, not been studied. Although highly speculative, it could be that the traces of Cr in the Fe_*x*_O subdomains have an enhancing effect on the AFM anisotropy, explaining the observation of *T*_N_ despite their small size.

Fig. 7(b) displays a ZFC magnetization curve recorded at 2 K exhibiting non-saturating behavior and high-field hysteresis, similar to what is frequently reported for Fe_*x*_O–Fe_3_O_4_ CS systems where the effect is attributed to uncompensated AFM interfacial magnetic moments and surface anisotropy. However, while the latter effect can be significant for fine and hollow particles with a large surface-to-volume ratio,^18^ they should have a diminishing influence on the magnetic response of the large particles in the present study. Here, the observed behavior is therefore assigned to the presence of uncompensated interfacial moments that experience competition between exchange interaction with the magnetically compensated part of the AFM phase, the Zeeman energy favoring an alignment with the external field and thermal fluctuations. However, while the effect mentioned above leads to a non-saturating behavior, the magnetization is reversible for fields above 5.5 T, see ESI.†

Cooling from room temperature to 2 K in a field of 1 T results in a significant horizontal shift of the hysteresis loop in the direction opposite to the direction of the cooling-field, Fig. 7(c). The shift can be explained by the proposed bimagnetic particle structure in Fig. 2 containing both AFM and FiM ordered phases. The Fe_*x*_O subdomains are paramagnetic at room temperature, and the spins can align with the applied field. When cooling below the Néel temperature, uncompensated interfacial spins can remain aligned with the field and the net FiM magnetization, while still being pinned by the compensated part of the AFM subdomains. The shift in hysteresis originates from the unidirectional exchange anisotropy induced by the pinning of interfacial moments along the field axis, making it more difficult to reverse the magnetization anti-parallel to the cooling field. As expected for an EB effect originating from the pinning of magnetic moments by the AFM subdomains, the observed horizontal shift vanishes around *T*_N_ (see Fig. S5†). The strength of the interfacial pinning depends on the AFM anisotropy, which directly scales with the volume of the Fe_*x*_O phase. Moreover, being an interfacial phenomenon, the exchange anisotropy relies on a large AFM–FiM interface area to maximize the effect. Consequently, large EB is usually detected in CS systems with significant Fe_*x*_O volume fraction, and the effect is reduced with decreasing core size due to a reduced AFM–FiM interface area and AFM anisotropy.^10,13,14^ The observed shift *μ*_0_*H*_E_ = 460 mT is one of the highest values reported for a Fe-oxide NP system^18^ and is quite remarkable given the small size and relatively low volume fraction of the Fe_*x*_O phase. With the presented results indicating that the subdomains have sufficient anisotropy to pin the FiM moments despite their small size, we attribute the large EB to the bimagnetic structure, which distributes the Fe_*x*_O phase throughout the particle volume, thereby increasing the surface to volume ratio and maximizing the interfacial area for a given AFM volume fraction. Interestingly, a comparable *H*_E_ was observed in 22 nm Fe_*x*_O–Fe_3_O_4_ CS cubes where the FiM phase was not only located at the surface, but also in the core of the nanocubes.^17^ These results indicate that the distribution of the two magnetic phases are important parameters for maximizing the EB effect. However, it should also be noted that distributing the AFM phase does not necessarily result in large *H*_E_, as evident from a recent study where Fe_3_O_4_ nanocubes with Fe_*x*_O subdomains exhibited only modest EB effects.^55^ The magnitude of *H*_E_ in the present study is also noteworthy considering the size of the particles, which with a diameter of ≈70 nm are ones of the largest reported in literature.^18,53^ It has been demonstrated that the *H*_E_ exhibits a non-monotonic dependence on the core size and shell thickness with a decreasing magnitude above a specific size.^7,56^ These results indicate that maximizing the EB effect relies on optimizing the relative dimensions of the two magnetic phases, which could become difficult as the size of a CS systems increases. Distributing the AFM phase by forming subdomains could thus have the advantage of allowing particle volume to increase without considerably changing the relative size of the two magnetic phases, thereby keeping the dimensions closer to that of smaller particles which typically exhibit more significant EB effects. Furthermore, *H*_E_ is critically influenced by the structural matching at the interface between the two phases.^7,57^ A lattice mismatch of only 1.4% is observed for the present system, which could be facilitating the exchange coupling across the AFM/FiM interface.

In addition to a large horizontal shift of the hysteresis loop, there is a 38% increase of the coercive field observed in the FC magnetization curve. The increase in coercivity can be explained by uncompensated spins not pinned to the AFM subdomains but rotating with the FiM phase through a spin drag effect.^19,20^ Observing a large *H*_E_ and significantly increased *H*_C_ indicates a substantial population of both pinned and unpinned interfacial moments, which can be understood given a distribution in size and thereby in the AFM anisotropy of the small subdomains.

## 4. Conclusions

In summary, we have demonstrated that NPs generated using spark ablation from stainless steel electrodes exhibit a novel bimagnetic structure composed of a 40 nm FiM (Fe,Cr)_3_O_4_ phase with 4 nm AFM Fe_*x*_O subdomains occupying up to 20% of the volume. Magnetization measurements demonstrate that the Fe_*x*_O subdomains exhibit an AFM ordering with sufficient anisotropy to produce a significant EB effect when cooled in a magnetic field across the Néel temperature. The observed EB field of *μ*_0_*H*_E_ = 460 mT is one of the highest values reported for a Fe_*x*_O–Fe_3_O_4_-based system and it is attributed to the formation of subdomains which maximizes the AFM–FiM area to produce a significant interfacial exchange coupling despite the relatively low amount of the Fe_*x*_O phase. These results indicate that the distribution of the AFM phase can significantly affect the EB and thus could provide a new parameter to explore for tuning the anisotropy in bimagnetic nanoparticles. Moreover, forming subdomains allows for large EB effects for small amounts of the AFM phase, which given the low magnetization of these materials, would be beneficial for exploring exchange-coupled bimagnetic systems for permanent magnet applications.

## Author contributions

RW and MEM conceived and designed the study. CB carried out the magnetic measurements and analysis (XMCD and SQUID), the multiplet simulations (CMT4XAS), the chemical composition study (XPS) and drafted the manuscript. CaP and MEM provided the NPs and the electron microscopy characterization. AK took part in the SQUID measurements and offered analysis insights. KMØJ provided the XRD data and analysis. EK took part in the XPS measurements and provided analysis insights. CiP took part in the XMCD measurements and provided help with the simulations. RW participated in the magnetometry and spectroscopy measurements and subsequent analysis. All authors commented on and contributed to writing the manuscript and have approved the final version.

## Conflicts of interest

There are no conflicts of interest to declare.

## Supplementary Material

NR-013-D1NR04614D-s001
