# Supplementary material for: Large exchange bias in Cr substituted Fe3O4 nanoparticles with FeO subdomains
Source: Nanoscale. 2021 Sep 8;13(37):15844–52. doi: 10.1039/d1nr04614d (PMC8485415; doi:10.1039/d1nr04614d)
Supplement: NR-013-D1NR04614D-s001 [file NR-013-D1NR04614D-s001.pdf]

Electronic Supplementary Material (ESI) for Nanoscale.
This journal is © The Royal Society of Chemistry 2021

Large exchange bias in Cr substituted Fe_3O_4 nanoparticles with FeO sub-domains - Supplementary

Claudiu Bulbucan,[a,b] Calle Preger,[b,c] Aram Kostanyan,[d,e] Kirsten M. Jensen,[f] Esko Kokkonen,[g] Cinthia Piamonteze,[e] Maria E. Messing,[a,b,c] and Rasmus Westerström[a,b]

1 XPS

The XPS data was measured at the SPECIES beamline[1] of MAX IV laboratory. Fig. S1 shows the room temperature 2p edges for both Fe and Cr at photon energies of 1150 and 970 eV, along with the background. As can be noted in the Fe 2p spectrum, there is no feature (satellite) at a binding energy of roughly 720 eV, which is indicative of the presence of both Fe^{2+} and Fe^{3+} ions[2,3].

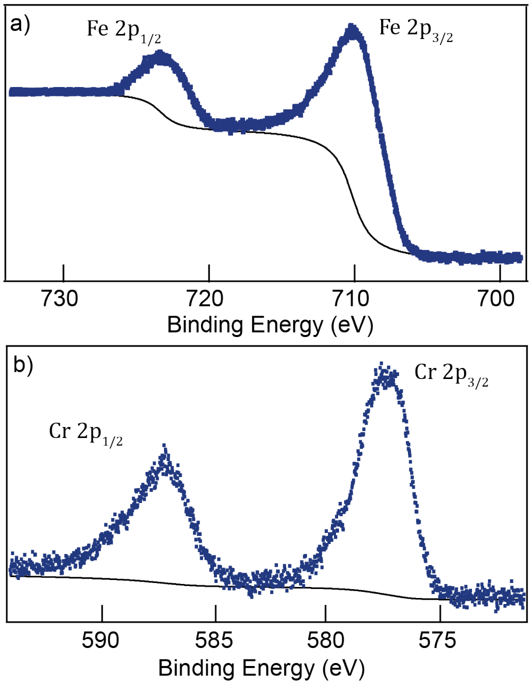

Fig. S 1 Room temperature $2p_{3/2,1/2}$ XPS spectra of a) Fe at a photon energy of 1150 eV and b) Cr at a photon energy of 970 eV.

[a] NanoLund, Lund University, Box 118, 22100 Lund, Sweden.
[b] Synchrotron radiation research, Lund University, SE-22100 Lund, Sweden; E-Mail: claudiu.bulbucan@sljus.lu.se
[c] Solid State Physics, Lund University, Box 118, 22100 Lund, Sweden.
[d] Physik-Institut, Universität Zürich, CH-8057 Zürich, Switzerland.
[e] Swiss Light Source, Paul Scherrer Institut, CH-5232 Villigen PSI, Switzerland.
[f] Department of Chemistry, Copenhagen University, Copenhagen, Denmark.
[g] MAX IV Laboratory, Lund University, PO Box 118, 221 00, Lund, Sweden.

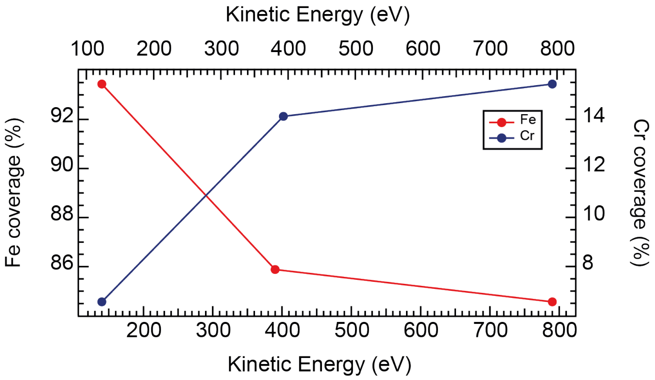

Fig. S 2 Fe and Cr coverage from the integrated XPS signal as a function of kinetic energy.

2 Cr simulations - charge transfer

The ground state of the Cr^{3+} ion consists of a mixture of $2p^63d^3$ and $2p^63d^4$ states whose energy difference can be written as $E(d^4) - E(d^3) = \Delta$, where E(d) represents the average configuration energy of the d multiplet, and a $\Delta = 0$ means a 50/50 mixture of the two states. The final state, where the core-hole potential is no longer negligible, can be written as $E(d^5) - E(d^4) = \Delta + (U_{dd} - U_{dc})$, where U_{dd} and U_{dc} are the correlation energy between the 3d electrons and the core-hole potential acting on the 3d electron, respectively. For 2p XAS calculations, only the difference ($U_{dd} - U_{dc}$) is relevant. The relation between the T hopping terms is assumed $T(t_{2g}) = -T(e_g)/2$[4]. The charge transfer parameters used in the present calculations are $(U_{dd} - U_{dc}) = -1$ eV and $T(e_g) = 2.8$ eV[5],

3 Minor-loop analysis

A magnetization curve that does not close at high applied fields could be an indicator of a minor-loop. This occurs when the applied field is not sufficiently strong as to "flip" all the moments in the sample along its direction. If the sample is cooled in an applied field, a minor loop may exhibit a shift of the hysteresis with respect to the field axis, similar to EB. The following analysis was derived from the work of Harres *et al*[6]

and it is aimed at showing that we measure major loops and the observed shift stems from the presence of EB.

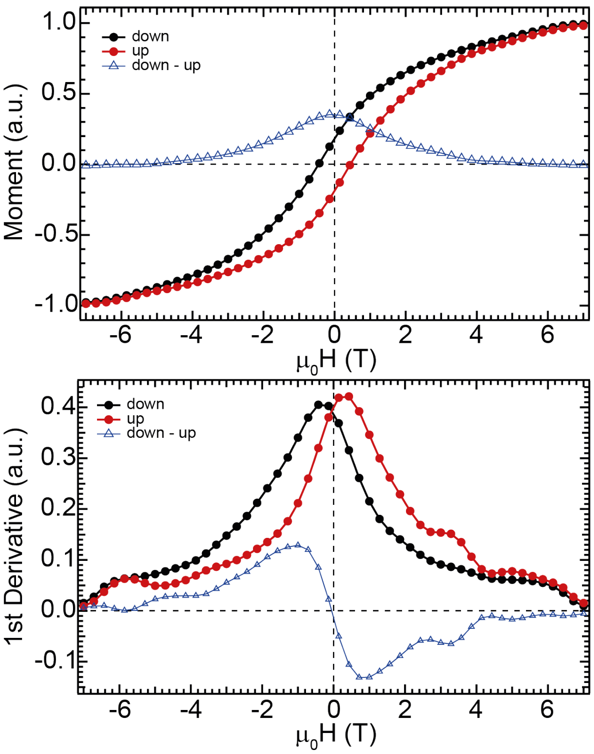

Fig. S 3 Top-part: Hysteresis loop with the two branches (i.e. when field goes down and up) separated and superimposed with their interpolations; the difference between branch_down – branch_up (to investigate where they overlap); bottom-part: the first derivatives of the two branches (over the whole field range) and the difference between the first derivatives (should be zero where the derivatives of the branches overlap).

The top-panel of Fig. S3 shows the 2K zero-field cooled data separated into two branches, namely branch_up and branch_down, names stemming from the field-sweep direction. The data is interpolated for ease of analysis. Furthermore, the difference between the two aforementioned branches is also shown and it reveals the absolute value of the field above which there is overlap, i.e. the hysteresis is closed. As can be observed, the difference goes to zero for fields of over 5.25 T within the error limit. For a finer analysis it is useful to look at the first derivatives of the two branches and the differences between them, shown in the bottom-panel of Fig. S3. A can be observed, the two branches coincide for fields higher than 5 T. However, the data is a bit noisy and so the difference between the two derivatives is expected to oscillate around the zero value.

4 Room temperature XMCD and simulations

The room temperature XMCD data and simulations are shown in Fig. S4. The best fit was achieved with 30% Fe_B^{2+}, 33% Fe_B^{3+} and 33% Fe_A^{3+}, yielding a Fe^{3+}:Fe^{2+} ratio of 2.4:1, in very good agreement to the ratio obtained from the XPS analysis.

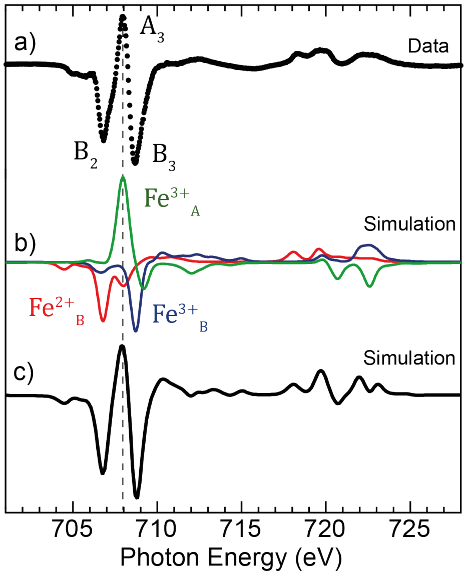

Fig. S 4 Room temperature data of a) the measured Fe *L*-edge XMCD spectrum; b) multiplet simulations for each Fe ion; c) weighted sum of the three Fe ions best reproducing the measured data.

The parameters used in the simulation were the same as for the 2 K Fe case (described in section 3.3 of the manuscript) with the exception of the temperature, which was set to 300 K. The increased presence of the divalent Fe ion at room temperature could be explained by the AFM to paramagnetic transition above the Néel temperature of $\approx$ 200 K.

5 Field cooled hysteresis

Fig. S5 shows three hysteresis curves recorded at 100, 150 and 200 K, with the measured H_E field values. As can be noted, the exchange field values decrease with increasing temperature, becoming vanishingly small when approaching the Néel temperature of the Fe_xO subdomains ($T_N \approx 210$ K). Although not precisely zero, the measured H_E at 200 K represents a 98% decrease compared to the value recorded at 2 K.

References

1 S. Urpelainen, C. Såthe, W. Grizolli, M. Agåker, A. R. Head, M. Andersson, S.-W. Huang, B. N. Jensen, E. Wallén, H. Tarawneh *et al.*, *Journal of*

This journal is © The Royal Society of Chemistry [year]

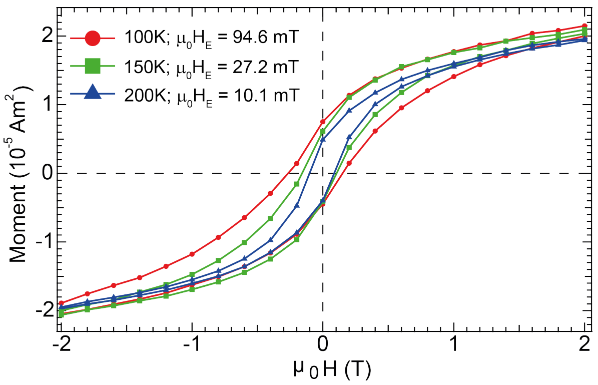

Fig. S 5 1T field cooled hysteresis curves at 100, 150 and 200 K. The EB field values are provided for each temperature.

synchrotron radiation, 2017, **24**, 344–353.

2 J. Deng, X. Wen and Q. Wang, *Materials Research Bulletin*, 2012, **47**, 3369–3376.

3 H. Yan, M. Zhang and H. Yan, *Journal of magnetism and magnetic materials*, 2009, **321**, 2340–2344.

4 K. Okada, A. Kotani and B. T. Thole, *Journal of electron spectroscopy and related phenomena*, 1992, **58**, 325–343.

5 T. Uozumi, *Doctoral Thesis*, University of Tokyo, 1997.

6 A. Harres, M. Mikhov, V. Skumryev, A. De Andrade, J. Schmidt and J. Geshev, *Journal of Magnetism and Magnetic Materials*, 2016, **402**, 76–82.
